# Alpha emitter radium-223 in patients with metastatic castration-resistant prostate cancer: A cost-utility analysis

**DOI:** 10.3389/fphar.2022.1003483

**Published:** 2022-10-21

**Authors:** Xiaohui Zeng, Qiao Liu, Chongqing Tan, Xiaomin Wan, Yunhua Wang, Xiaowei Ma

**Affiliations:** ^1^ Department of Nuclear Medicine/PET Image Center, The Second Xiangya Hospital of Central South University, Changsha, China; ^2^ Department of Pharmacy, The Second Xiangya Hospital of Central South University, Changsha, China

**Keywords:** cost effectiveness, radium-223, metastatic castration-resistant prostate cancer, QALY, China

## Abstract

**Objective:** To assess the cost effectiveness of radium-223 dichloride for patients with metastatic castration-resistant prostate cancer (mCRPC) in China.

**Materials and methods:** A Markov model was developed to estimate the long-term health and economic outcomes of radium-223 plus best standard care (BSC) treatment and BSC only for bone mCRPC patients over a lifetime horizon. The patients and interventions were modeled according to the ALSYMPCA trial. Costs were collected from a Chinese health system perspective. Utility values were derived from the published literature. The base-case model results were quality-adjusted life year (QALY), total cost, and incremental cost-utility ratio (ICUR). Uncertainty analyses were performed to assess the robustness of our conclusions.

**Results:** Compared with the BSC arm, radium-223 achieved an excess 0.344 QALYs with an incremental cost of $29,459, resulting in an ICUR of $85,647 per QALY. The probability of Ra-223 being cost effective for the patients with bone mCRPC was sharply low (<0.5%) at a willingness-to-pay threshold of $38,136/QALY. Uncertainty analyses revealed that the model is robust to all the input parameters.

**Conclusion:** Radium-223 is unlikely to be cost effective in patients with bone mCRPC at the current WTP threshold, from a Chinese health system perspective. In affluent areas with a high per-capita GDP, radium-223 therapy may be cost effective.

## Introduction

Prostate cancer is the most commonly diagnosed cancer and the second leading cause of death among males, with an estimated 1,414,259 new cases and 375,304 fatalities worldwide (Sung et al., 2021; Siegel et al., 2022). The majority of advanced prostate cancers eventually stop responding to the standard androgen deprivation treatment (ADT), which is categorized as castration-resistant prostate cancer (CRPC). Up to 90% of patients with metastatic CRPC (mCRPC) will develop bone metastases (Parker et al., 2013). Clinical practice guidelines from the European Society for Medical Oncology (ESMO), Chinese Society of Clinical Oncology (CSCO), and American Society of Clinical Oncology (ASCO) all recommend the use of abiraterone, enzalutamide, cabazitaxel, and radium-223 dichloride (for men with bone-predominant, symptomatic mCRPC without visceral metastases) in patients with mCRPC who are ineligible for docetaxel (Guidelines of Chinese Society of Clinical Oncology, 2020; Parker et al., 2020; Saylor et al., 2020). According to the ASCO and CSCO guidelines, Sipuleucel-T may be offered to mCRPC with asymptomatic/minimally symptomatic (Guidelines of Chinese Society of Clinical Oncology, 2020; Saylor et al., 2020); and palliative care should be offered to all patients (Guidelines of Chinese Society of Clinical Oncology, 2020; Parker et al., 2020; Saylor et al., 2020). Bisphosphonate or denosumab is recommended as palliative care for patients with bone mCRPC at risk for clinically significant skeletal-related events, and external beam radiotherapy treatment (EBRT) is recommended for palliation of painful, uncomplicated bone metastasis (Guidelines of Chinese Society of Clinical Oncology, 2020; Parker et al., 2020; Saylor et al., 2020). According to the CSCO guidelines, the best standard of care (BSC) includes alleviating pain, nutritional support, psychotherapy, and preventing skeletal-related events (bisphosphonate, EBRT, etc.) (Guidelines of Chinese Society of Clinical Oncology, 2020).

Bone disease and its complication are major causes of death for patients with prostate cancer and have imposed a substantial economic burden and decreased the quality of life (Lange and Vessella, 1998; Roodman, 2004; Hagiwara et al., 2013). Therefore, prevention and delay of the symptomatic skeletal event (SSE) are vital in the management of patients with mCRPC. Denosumab, bisphosphonates, and radioisotopes are among the treatments that can relieve pain and postpone SSE but do not increase survival (Finlay et al., 2005; Lipton, 2010; Fizazi et al., 2011; Sartor et al., 2011). Conversely, available effective therapies including docetaxel, cabazitaxel, abiraterone, and enzalutamide that have been proven to improve mCRPC patients’ survival have not been demonstrated to prevent against SSE(Berthold et al., 2008; de Bono et al., 2010; de Bono et al., 2011; Penson et al., 2016).

Radium-223 dichloride (Ra-223), as a bone-targeted alpha therapy, is a first-in-class radiopharmaceutical that has been shown to have both survival and SSE benefit for CRPC with symptomatic bone metastases (bone mCRPC) (Kluetz et al., 2014), based on the findings of a double-blind, randomized phase 3 ALSYMPCA trial (Parker et al., 2013; Hoskin et al., 2014; Sartor et al., 2014). The ALSYMPCA trial demonstrated that compared with the best standard of care (BSC), Ra-223 significantly prolonged overall survival (median 14.9 months vs. 11.3 months; hazard ratio [HR] 0.70, 95% CI 0.58–0.83) and the time to first SSE (median 15.6 months vs. 9.8 months; HR 0.66, 95% CI 0.52–0.83) for patients with bone mCRPC. Another double-blind randomized phase 3 trial (ERA-223) found that adding Ra-223 to abiraterone plus prednisone or prednisolone did not improve SSE in patients with bone mCRPC, and was associated with a higher risk of bone fractures than adding placebo (Smith et al., 2019). An interim safety analysis of a global, prospective, non-interventional study (REASSURE) indicated that the short-term safety profile of Ra-223 in routine clinical practice was comparable to other clinical studies, irrespective of prior chemotherapy treatment (Dizdarevic et al., 2019).

Due to its superior efficacy, Ra-223 is recommended as category 1 for bone mCRPC in clinical guidelines (Guidelines of Chinese Society of Clinical Oncology, 2020; Nation Comprehensive Cancer Network, 2022), and was approved by the Chinese National Medical Products Administration (NMPA) in 2020. Several studies have evaluated the economic impact of Ra-223 in patients with bone mCRPC in different countries, including Spanish, Ireland, England and Wales, Canada, and Dutch (Tirado Mercier et al., 2018; National Centre for Pharmacoeconomics (NCPE), 2022; National Institute for Health and Care Excellence (NICE), 2022; Canadian Agency for Drugs and Technologies in Health (CADTH), 2016; Peters et al., 2018). However, it is unclear whether Ra-223 is cost effective for Chinese patients with bone mCRPC. Therefore, the aim of this study is to evaluate the cost effectiveness of Ra-223 in the treatment of patients with bone mCRPC from a Chinese health system perspective.

## Materials and methods

### Methodological design

To meet the aim described above, a cost-utility analysis was carried out to estimate the long-term health and economic outcomes of Ra-223 plus BSC and placebo plus BSC for bone mCRPC patients. A Markov model was developed in TreeAge Pro software (TreeAge Software, Williamstown, MA), and the model simulation outcomes were presented as total costs, quality-adjusted life years (QALYs), and incremental cost-utility ratio (ICUR). All costs and utilities were discounted to 2021 using a 5% annual rate according to China Guidelines for Pharmacoeconomic Evaluations (2020) (Certified Public Accountant, 2020) and an average exchange rate in December 2021 of 1 US dollar (USD) to 6.37 Chinese Yuan (RMB) (The People’s Bank of China, 2022). The willingness-to-pay (WTP) threshold of $38,136/QALY, which equals to three times the per-capita gross domestic product (GDP) of 2021 (National Bureau of Statistics of China, 2022), was used in the cost-utility analysis based on the recommendation of the China Guidelines for Pharmacoeconomic Evaluations.

### Patients and intervention

The patients and intervention were modeled according to the ALSYMPCA trial, which verified a superior benefit of Ra-223 plus BSC among mCRPC patients, who previously received treatment of docetaxel or were unsuitable for docetaxel, with two or more bone metastases and no known visceral metastases (Parker et al., 2013). In brief, 921 patients were randomly assigned (2:1) to receive the therapies of Ra-223 (50 kBq/kg, once per 4 weeks, 6 intravenous injections) plus BSC or placebo plus BSC. The BSC treatments were administered to both arms, including local external beam radiation, glucocorticoids, antiandrogens, ketoconazole, estramustine, or diethylstilbestrol.

### Model structure

**FIGURE 1:**
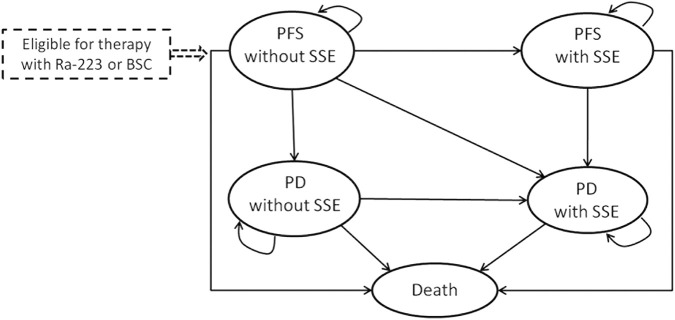
Markov model for patients with metastatic castration-resistant prostate cancer. PFS: progression-free survival; SSE: symptomatic skeletal event; Ra-223: radium-223; PD: progression survival; BSC: best standard care.

A Markov model was established to estimate the costs and health efficacy of treatment with Ra-223 plus BSC versus BSC alone in patients with bone mCRPC (Figure 1). There are five mutually exclusive health states in the model: progression-free survival (PFS) without SSE, progression (PD) without SSE, PFS with SSE, PD with SSE, and death. In a nutshell, all eligible patients enter the health state “PFS without SSE,” and receive Ra-223 plus BSC treatment (Ra-223 arm) or placebo plus BSC treatment (BSC arm). Patients can move from one state to another or stay in their initial health state at the end of each Markov cycle. The Markov model cycle length was chosen to be 4 weeks, mirroring the Ra-223 therapy cycle.

The main variables that determine the Markov transition probabilities for the BSC arm were derived from the ALSYMPCA study (Parker et al., 2013). The standard techniques were used to perform extrapolation of the time to death, the time to SSE, and the time to disease progression (Guyot et al., 2012). The first step was to reconstruct the individual patient-level data (IPD) using published survival curves, the number of patients at risk, and the total events. Second, the four common survival models—exponential, Weibull, log-normal, and log-logistic—were tested using the reconstructed IPD. Finally, the Akaike information criterion, visual inspection, and statistical criteria were used to determine that the log-logistic function should be used for the current economic evaluation. Supplementary Appendix SA1 contains specifics regarding the selection of survival distributions. The hazard ratios (HRs) published in the clinical ALSYMPCA were used to calculate the transition probabilities for the Ra-223 arm. The variables of the log-logistic function and the HRs are displayed in Table 1.

**TABLE 1:** Model input parameters estimated or derived from the clinical trial.

Variable	Base value	Range	Distribution
Log-logistic OS survival model with BSC arm^a^	Theta = 0.0890; Kappa = 1.9439	—	—
Log-logistic SSE survival model with BSC arm^a^	Theta = 0.1003; Kappa = 1.3675	—	—
Log-logistic PFS survival model with BSC arm^a^	Theta = 0.4778; Kappa = 2.7054	—	—
OS hazard ratio Ra-223 arm vs. BSC arm Parker et al., 2013	0.70	0.58–0.83	Log-normal
SSE hazard ratio Ra-223 arm vs. BSC arm Parker et al., 2013	0.66	0.52–0.83	Log-normal
PFS hazard ratio Ra-223 arm vs. BSC arm Parker et al., 2013	0.64	0.55–0.75	Log-normal
Risks for adverse events (%)			
Anemia in Ra-223 arm Parker et al., 2013	12.67	8.87–16.47	Beta
Anemia in BSC arm Parker et al., 2013	12.96	9.07–16.84	Beta
Thrombocytopenia in Ra-223 arm Parker et al., 2013	6.33	4.43–8.23	Beta
Thrombocytopenia in BSC arm Parker et al., 2013	1.99	1.40–2.59	Beta
Fatigue in Ra-223 arm Parker et al., 2013	4.00	2.80–5.20	Beta
Fatigue in BSC arm Parker et al., 2013	5.98	4.19–7.77	Beta
Bone pain in radium-223 arm Parker et al., 2013	20.83	14.58–27.08	Beta
Bone pain in BSC arm Parker et al., 2013	25.58	17.91–33.26	Beta
Spinal-cord compression in Ra-223 arm Parker et al., 2013	3.33	2.33–4.33	Beta
Spinal-cord compression in BSC arm Parker et al., 2013	5.65	3.95–7.34	Beta
Mean number of injections Ra-223 Parker et al., 2013; Guidelines of Chinese Society of Clinical Oncology, (2020)	6.00	5.87–6.13	Normal
Proportion of bisphosphonate given in Ra-223 arm (%) Sartor et al., 2014	42.67	29.87–55.47	Beta
Proportion of bisphosphonate given in BSC arm (%) Sartor et al., 2014	43.00	30.10–55.90	Beta
Proportion of EBRT in Ra-223 arm (%) Sartor et al., 2014	30.29	21.21–39.38	Beta
Proportion of EBRT in BSC arm (%) Sartor et al., 2014	34.20	23.94–44.46	Beta

^a^ Estimated from the ALSYMPCA study; OS, overall survival; SSE, symptomatic skeletal event; PFS, progression-free survival; BSC, best standard of care; Ra-223: radium-223; EBRT, external beam radiotherapy treatment.

According to the China Guidelines for Pharmacoeconomic Evaluations, the cost-utility analysis of the current model was performed from a Chinese health system perspective using a lifetime horizon and a half-cycle correction.

### Inputs used to populate the model

Medical costs of health resources were collected in accordance with the clinical practice of the ALSYMPCA trial. Direct medical costs were calculated in terms of standard procedure for Ra-223 therapy, management of serious adverse events (AEs), BSC (bisphosphonates and local external beam radiation), routine follow-up care (prostatic specific antigen testing, physician examination, imaging CT scan, complete blood count, bone scan, and testosterone levels test), subsequent lines treatment in progression state, and terminal care. Supplementary Appendix SA2 offered information regarding the frequency of health services. The clinical trial’s mean number of Ra-223 injections was used to calculate the drug’s total costs. Only grade 3/4 AEs with a frequency greater than 3% were taken into account to estimate the AEs costs in the current economic evaluation. Risks of bisphosphonate and external beam radiotherapy were multiplied by their unit costs as the BSC cost. Costs of tumor-related orthopedic surgical and pathological fractures were not estimated in our analysis because of their identical incidences and little healthcare spending. Because zoledronic acid was the most commonly used bisphosphonate in the ALSYMPCA trial, we assumed it was available when assessing the cost of bisphosphonate. Routine follow-up care was given according to the Chinese clinical guidelines for the management of prostate cancer (Guidelines of Chinese Society of Clinical Oncology, 2020). All model input parameters estimated or derived from the clinical trial are shown in Table 1.

Based on Chinese experts’ opinion, all patients with progression switched to abiraterone acetate and prednisone as subsequent lines of treatment after 4 weeks of the last injection of Ra-223. The risks of the subsequent lines of treatment in the two arms were obtained from another economic evaluation (Tirado Mercier et al., 2018). Unit costs, shown in Table 2, were derived from published studies (Zeng et al., 2012; Hu et al., 2019; Zeng et al., 2019; Qin et al., 2021), the site of the big data service platform for China’s health industry (yaozh, 2022), or estimated according to local charges and adjusted by a clinical physician from a Chinese health system perspective.

**TABLE 2:** Unit costs, utilities, and other parameter inputs used in the model.

Variable	Base value	Range	Distribution
Unit cost ($)			
Radium-223, per unit^a^	3139.72	2197.80–4081.64	Gamma
Zoledronic acid, per unit; yaozh (2022)	76.30	53.41–99.19	Gamma
Abiraterone, per mg; Hu et al. (2019)	0.091	0.064–0.118	Gamma
Prednisone, per mg; Hu et al. (2019)	0.21	0.147–0.273	Gamma
Anemia, per incidence; Hu et al. (2019)	42.65	29.86–55.45	Gamma
Thrombocytopenia, per incidence; Hu et al. (2019)	552.28	386.60–717.96	Gamma
Fatigue, per incidence; Hu et al. (2019)	84.84	59.39–110.29	Gamma
Bone pain, per incidence; Hu et al. (2019)	91.52	64.07–118.98	Gamma
Spinal-cord compression, per incidence; Hu et al. (2019)	193.88	135.72–252.04	Gamma
External beam radiation, per unit^a^	1883.83	1,318.68–2448.98	Gamma
End-of-life treatment, per patient; Qin et al. (2021)	3311.30	2317.91–4304.69	Gamma
Prostatic specific Antigen testing, per time; Qin et al. (2021)	22.84	15.99–29.69	Gamma
Physician examination, per time^a^	54.95	38.47–71.44	Gamma
Imaging CT scan (abdominal), per time; Zeng et al. (2012)	59.00	41.30–76.70	Gamma
Complete blood count, per time^a^	3.14	2.20–4.08	Gamma
Bone scan, per time; Zeng et al. (2019)	87.00	69.60–104.40	Gamma
Testosterone levels test, per time^a^	7.85	5.50–10.21	Gamma
Utilities; Tirado Mercier et al. (2018)			
Progression-free without SSE in radium arm	0.617	0.588–0.645	Beta
Progression-free without SSE in BSC arm	0.554	0.492–0.616	Beta
Progression-free with SSE	0.475	0.444–0.506	Beta
Progression without SSE	0.511	0.453–0.568	Beta
Progression with SSE	0.474	0.443–0.505	Beta
Other parameters			
Risk of subsequent lines treatment in Ra-223 arm; Tirado Mercier et al. (2018)	53.70%	37.59%–69.81%	Beta
Risk of subsequent lines treatment in BSC arm; Tirado Mercier et al. (2018)	62.70%	43.89%–81.51%	Beta
Discount rate; Certified Public Accountant (2020)	5%	1%–8%	Fixed in PSA

^a^ Estimated by local charge and adjusted by a physician; SSE, symptomatic skeletal event; BSC, best standard of care; Ra-223: radium-223; PSA, probabilistic sensitivity analysis.

Utility values were obtained from published literature (Tirado Mercier et al., 2018) and displayed in Table 2. The utility values were different between patients treated with Ra-223 and BSC. Once the disease progressed, the same utility values were assumed for both arms.

### Uncertainty analyses

We conducted a series of uncertainty analyses to evaluate the robustness of the model results. In one-way sensitivity analysis, model input parameters were varied within their ranges to explore the influence of each individual parameter on the results. The ranges of all parameters were obtained from the relevant derivation or ±30% of the base values. Monte Carlo simulations of 1,000 iterations were performed in a probabilistic sensitivity analysis by setting specific patterns of distribution for each parameter. All ranges and distributions selected for uncertainty analyses were listed in Table 1 and Table 2. Additionally, considering the survival curves used to model the survival curves may impact results greatly, we performed a scenario analysis using different fitted survival curves.

## Results

### Base-case results

Patients treated with Ra-223 achieved QALYs of 1.047, which was 0.344 more than those receiving BSC treatment. The total cost per patient in the Ra-223 and BSC arms was 54,963 USD and 25,504 USD, respectively. The ICUR for the Ra-223 arm versus the BSC arm was $85,647 per QALY (Table 3).

**TABLE 3:** Base-case model results.

Group	QALYs	Cost ($)	ICER ($/QALY)
BSC arm	0.703	25,504	
Radium-223 arm	1.047	54,963	
Incremental	0.344	29,459	85,647

BSC, best standard of care; QALY, quality-adjusted life year; ICER, incremental cost-effectiveness ratio.

### Sensitivity analyses results

**FIGURE 2:**
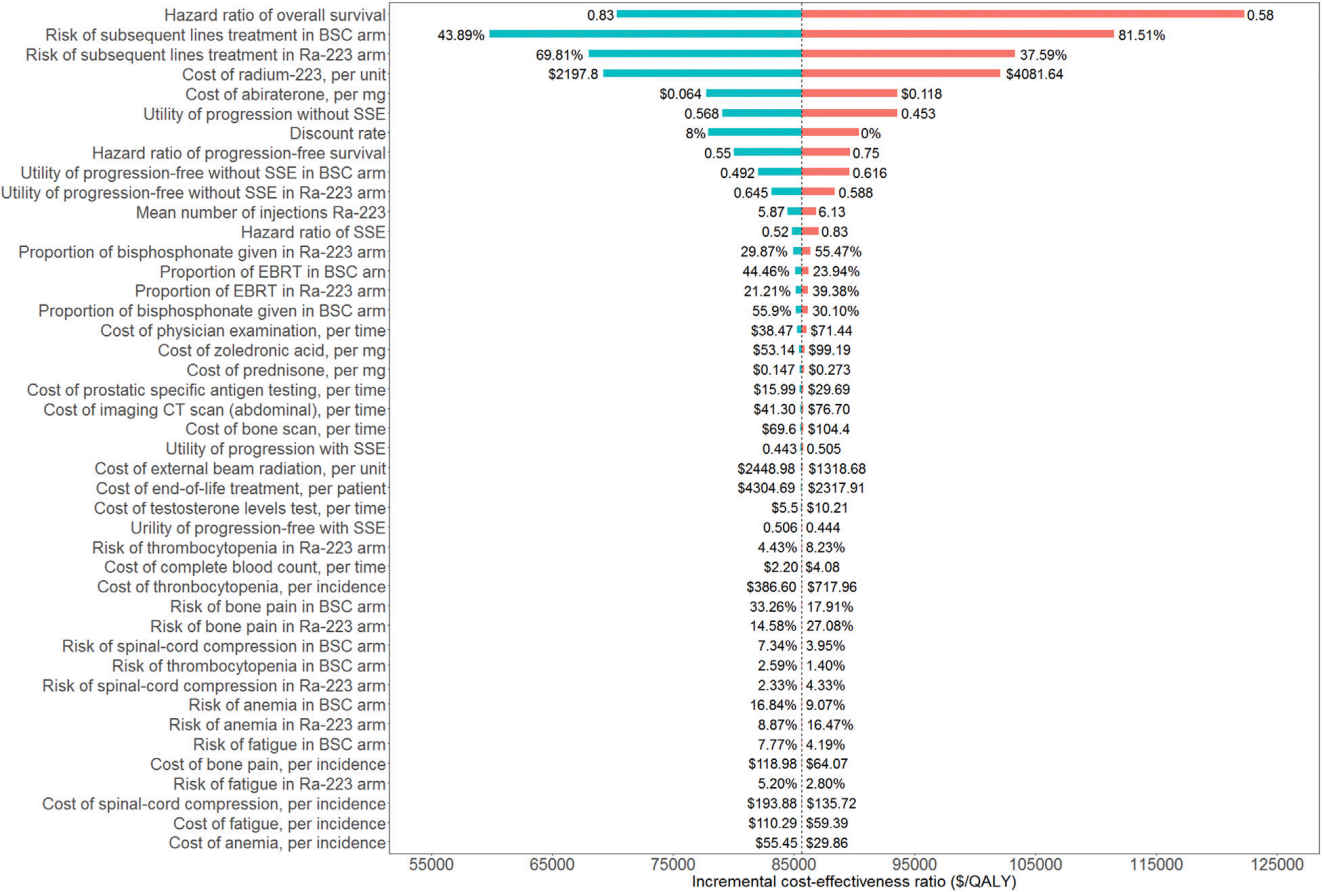
Tornado diagram of one-way sensitivity analyses. BSC: best standard care; Ra-223: radium-223; SSE: symptomatic skeletal event; EBRT: external beam radiation treatment.

A tornado diagram was used to show how the one-way sensitivity analyses turned out (Figure 2). The ICUR was most affected by the overall survival HR. Other sensitive parameters were risks of subsequent lines of treatment in both groups, the cost of radium-223 and abiraterone, the utility of progression without SSE, and the discount rate. All of the model’s input parameters failed to result in an ICUR below the WTP threshold of $38,136/QALY.

**FIGURE 3:**
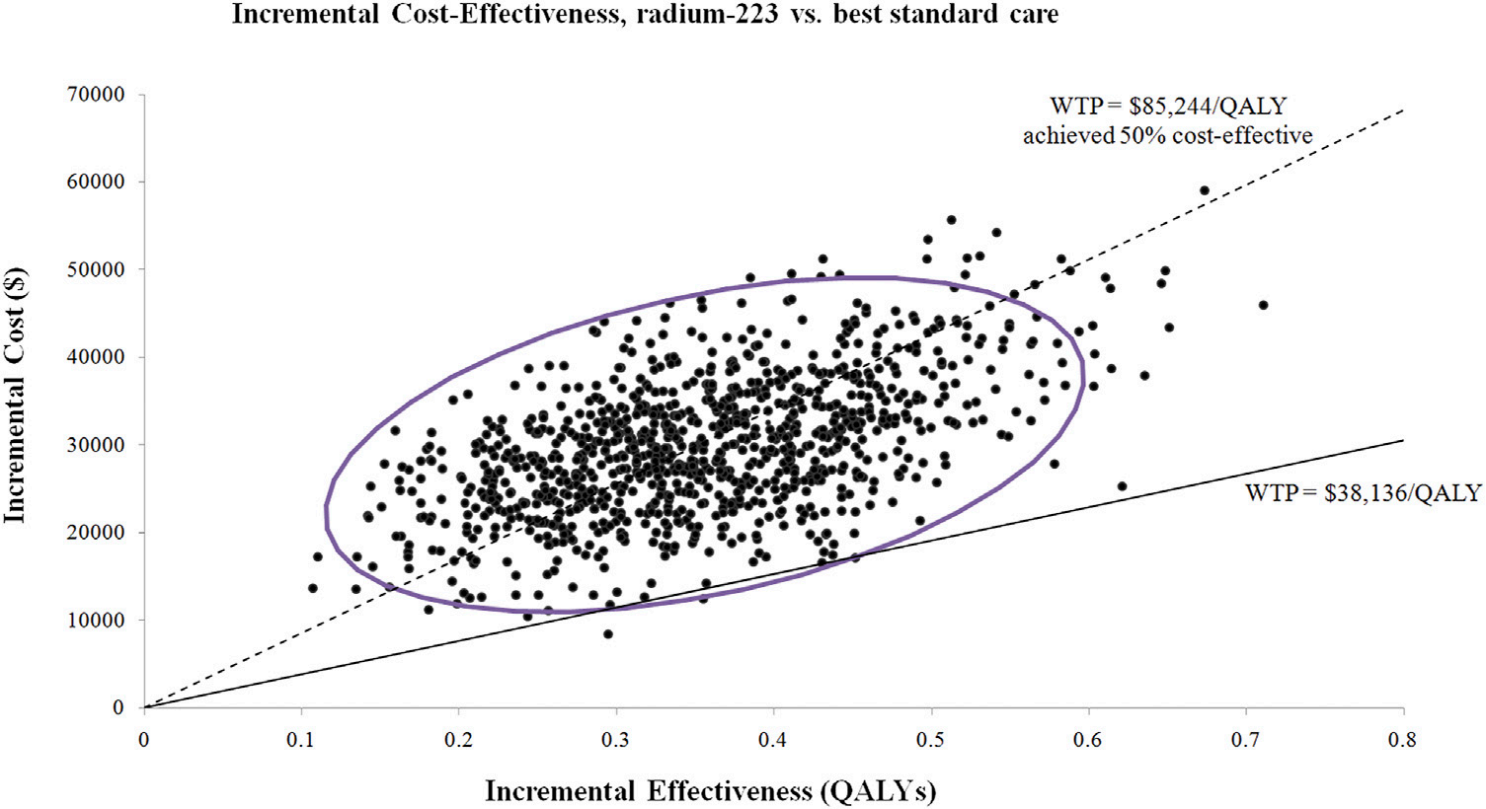
Incremental cost-effectiveness scatterplot of 1,000 iterations. WTP: willingness to pay; QALY: quality-adjusted life years.

**FIGURE 4:**
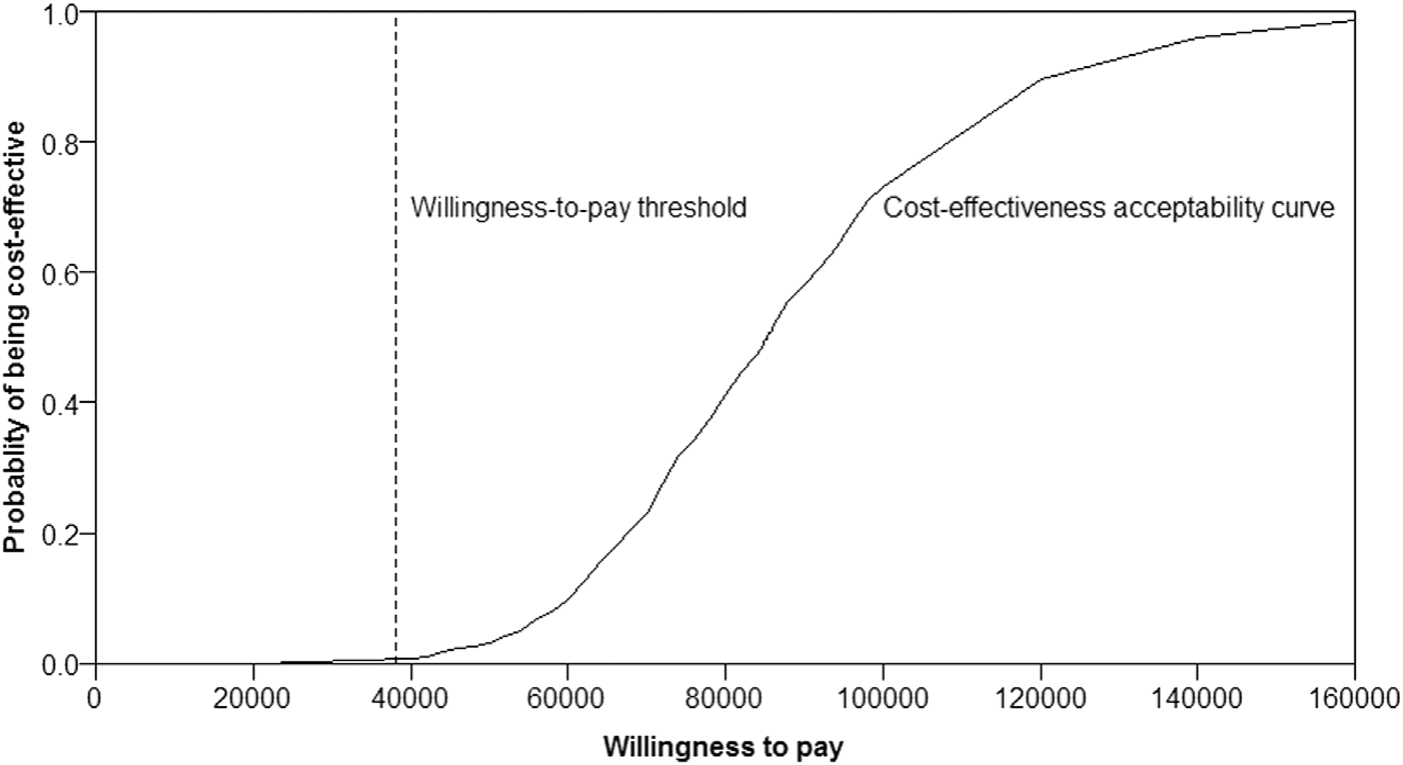
Acceptability curve of radium-223 being cost effective.

Almost the majority of the dots in the incremental cost-effectiveness scatterplot of 1,000 iterations were above the WTP threshold of $38,136/QALY (Figure 3). If the WTP threshold was more than $85,824/QALY, more than 50% of dots were cost-effective (below the WTP threshold). The probability of Ra-223 being cost-effective for the patients with bone mCRPC was sharply low (<0.5%) at the current WTP threshold ($38,136/QALY), according to the cost-effectiveness acceptability curve (Figure 4).

Scenario analyses using different fitted survival curves were displayed in Table 4. Although different fitted survival distributions impacted the results greatly, all of them did not result in the ICER below the WTP threshold ($38,136/QALY).

**TABLE 4:** Scenario analyses using different fitted survival curves.

Fitted survival distribution	Group	QALY	Cost ($)	ICUR ($/QALY)
Exponential	BSC arm	0.691	24,819	—
	Radium-223 arm	0.980	52,495	95,765
Weibull	BSC arm	1.706	68,581	—
	Radium-223 arm	2.126	109,525	97,486
Log-normal	BSC arm	0.689	25,083	—
	Radium-223 arm	0.993	53,879	94,724

BSC, best standard of care; QALY, quality-adjusted life year; ICER, incremental cost-utility ratio.

## Discussion

Ra-223 has been shown to be effective for treating patients with bone mCRPC in the ALSYMPCA trial due to its prolongation of overall survival and the first time to SSE (Parker et al., 2013; Hoskin et al., 2014; Sartor et al., 2014). However, after receiving NMPA approval, it is also necessary to assess its economic impact. In the current study, from a Chinese health system perspective, we evaluated the lifetime horizon costs and effectiveness associated with Ra-223 plus BSC and BSC only for bone mCRPC using a Markov model. The efficacy and safety data were taken from the ALSYMPCA trial, and the cost was collected from the published literature, or estimated according to local charges and adjusted by clinical physicians, on the basis of the clinical practice and the Chinese clinical guidelines. The result of the base-case analysis demonstrated that the ICUR was unfavorable at $85,647 per QALY gained, which was higher than the WTP threshold of $38,136/QALY.

The outcomes of our investigation were robust to all of the input parameters, according to the uncertainty analyses. Although the hazard ratio of the overall survival, risk of subsequent lines of treatment in both groups, and cost of radium-223 impacted the ICUR materially, none of them led to the ICUR lowering the WTP threshold. According to the probabilistic sensitivity analyses, adding Ra-223 to the BSC for patients with bone mCRPC exceeds the current WTP threshold. Only if the WTP threshold is more than $85,824/QALY will the Ra-223 therapy have a greater than 50% chance of being cost-effective. Of note, in the Chinese mainland, there are 31 province-level administrative divisions, in which the per-capita GDP differs significantly. The per-capita GDP ranged from $6,421 (Gansu province) to $28,870 (Beijing city) in 2021 (National Bureau of Statistics of China, 2022). Obviously, the Ra-223 treatment would be a more cost-effective alternative if we used Beijing’s per-capita GDP as the recommended WTP threshold (3 × $28,870 = $86,610) because the probability of Ra-223 being cost-effective is more than 50% when the WTP threshold is higher than $86,610/QALY (Figure 3).

Our research represents, as far as we are aware, the first economic evaluation of Ra-223 from the Chinese health system perspective. We believe that the current findings can offer crucial information to Chinese decision makers, especially the National Healthcare Security Administration and the medical resource payers, as well as other payers of medical resources.

There is another published article that evaluated the cost effectiveness of Ra-223 on the basis of the ALSYMPCA trial, which was performed from the perspective of the Spanish national health system (Tirado Mercier et al., 2018). When compared to BSC treatment alone, Ra-223 in their study achieved an incremental QALYs of 0.35 and a cost of €9,631, and the ICUR was €27,606/QALY. The incremental cost and the ICUR were not comparable between that study and our study because various countries have distinct national characteristics. But our study’s incremental QALYs of 0.344 is comparable to the earlier finding (0.35). An economic evaluation, from the Dutch societal perspective, was conducted to investigate the cost effectiveness of Ra-223 compared with abiraterone, cabazitaxel, and enzalutamide, in patients with mCRPC previously treated with docetaxel (Peters et al., 2018). In that study, compared with cabazitaxel and abiraterone, Ra-223 resulted in €4,465 and €6,092 lower costs and 0.01 and 0.02 higher QALYs, separately, and compared with enzalutamide, Ra-223 achieved slightly lower QALYs (−0.06) and €7,390 lower costs. At the informal WTP threshold of €80,000/QALY, Ra-223 revealed a cost-effective chance with 64%, 61%, and 54%, compared with abiraterone, enzalutamide, and cabazitaxel, respectively. Therefore, they concluded that Ra-223 maybe a less costly strategy compared with abiraterone, cabazitaxel, and enzalutamide.

Some health technology assessment (HTA) reports assessed the cost effectiveness of Ra-223 for patients with mCRPC. The National Centre For Pharmacoeconomics (NCPE) calculated the ICUR of Ra-223 versus BSC with €79,948/QALY (National Centre for Pharmacoeconomics (NCPE), 2022). As such, Ra-223 was deemed not cost effective at a WTP threshold of €45,000/QALY. The NCPE also discussed a company evaluation of Ra-223 versus abiraterone from an indirect comparison analysis and inferred that the probability of cost effectiveness of Ra-223 versus abiraterone was 41% (National Centre for Pharmacoeconomics (NCPE), 2022). The National Institute for Health and Care Excellence (NICE) noted that the ICUR for Ra-223 versus BSC was likely above £50,000/QALY, which is above the normal WTP threshold of £20,000-£20,000 per QALY gained (National Institute for Health and Care Excellence (NICE), 2022). Consequently, the NICE concluded that Ra-223 could not be considered a cost-effective use of national health services (NHS) resources in England and Wales, Ra-223 is recommended only when a negotiated discounted price is reached. In Canada, the Canadian Agency for Drugs and Technologies in Health (CADTH) reviewed the economic evaluation of Ra-223 for patients with bone mCRPC and suggested that Ra-223 was not cost-effective (Canadian Agency for Drugs and Technologies in Health (CADTH), 2016).

Our study has a number of strengths. First, as mentioned above, our study was the first economic evaluation of Ra-223 in China, which can provide Chinese decision makers with crucial data. Second, a large multicentre phase III trial with 921 patients, including Chinese patients, served as the basis of the current study’s model analyses (Parker et al., 2013). The randomized controlled trial is deemed to be the most scientific method and provides the most rigorous evidence for determining the net benefit of a new drug or a therapy procedure. Third, in order to best depict Chinese conditions, the health resources we employed in the model were collected from Chinese published literature or estimated by local charges and adjusted by a physician associated with the clinical practice and the Chinese clinical guidelines. Last but not the least, to test the impact of each input parameter’s certainty on the outcome, the model’s input parameters were varied within the appropriate ranges and defined patterns of distribution. All uncertainty analyses revealed that the results we estimated were robust regardless of how the parameters varied.

There are some limitations in our study to be noted. First off, only grade 3/4 AEs with a percent greater than 3% were taken into account when estimating the AEs costs, which might result in uncertainty about the management costs of AEs. However, the economic evaluation focused on estimating ICUR between the two arms, and the incremental total cost was used to calculate the ICUR. Therefore, the AEs with lower risk would only have a modest impact on the final result. On the other hand, there would be less doubt if patients with grade 1/2 AEs rarely received extra treatment. Last, the findings of the one-way sensitivity analyses showed that the ICUR was not sensitive to the AEs cost and the relative incidence (Figure 2). As with any modeling study, there is an inherent limitation to utilizing the log-logistic function to extrapolate survival curves beyond the time horizon of the clinical trial. The usage of BSC as an additional active treatment is the last limitation. In China, abiraterone ought to be an alternative treatment for those with bone mCRPC. However, currently, there was not yet a clinical trial that directly compared the Ra-223 to abiraterone. We did not use the indirect comparison methods since they should take into account how comparable clinical trials are to one another and would undermine the validity of the current constructed model. We will update our results when suitable clinical data are available. Despite the limitations listed above, this study will nevertheless be helpful to physicians, payers, and decision makers in China as it is the first economic evaluation of Ra-223 from a Chinese perspective.

In conclusion, from the Chinese health system perspective, adding radium-223 to the best standard care is unlikely to be cost effective, in patients with bone metastatic castration-resistant prostate cancer at the current WTP threshold. However, in affluent regions with high per-capita GDP, radium-223 therapy may be cost effective.

## Data Availability

The original contributions presented in the study are included in the article/Supplementary Material; further inquiries can be directed to the corresponding authors.
