# Supplementary material for: Alpha emitter radium-223 in patients with metastatic castration-resistant prostate cancer: A cost-utility analysis
Source: Front Pharmacol. 2022 Oct 21;13:1003483. doi: 10.3389/fphar.2022.1003483 (PMC9633991; doi:10.3389/fphar.2022.1003483)
Supplement: Supplementary file 1 [file DataSheet1.docx]

**Supplementary Appendix 1:** **The selection of survival distributions for best standard of care arm**

First, the individual patient-level data (IPD) were reconstructed on the basis of published survival curves, number of patients’ at risk, and the total events. Second, the reconstructed IPD were applied to test the four common survival models: Exponential, Weibull, Lognormal, and Log-logistic. Finally, we selected the optimal survival function based on Akaike information criterion (AIC), visual inspection and statistical criteria.

**Overall Survival Fit**

As for the overall survival (OS) Kaplan-Meier curves of best standard of care (BSC) arm, the visual fits of the Four mentioned-above parametric survival distributions was illustrated in **Figure S1A**, and the AIC statistics were detailed in **Table S1**. The visual fits showed that these four distributions provided similar fits for published OS data. Based on the AIC statistics, the log-logistic distribution may be appropriate as it provided the lowest AIC value.

**Table S1: AIC statistics for survival curves data of BSC arm**

| AIC value | Parametric survival distribution | | | |
| --- | --- | --- | --- | --- |
|  | Exponential | Weibull | Log-normal | Log-logistic |
| Overall survival | 1487.547 | 1455.887 | 1444.190 | 1443.498 |
| Time to SSE | 876.1124 | 877.0147 | 860.5323 | 864.3951 |
| Progression-free survival | 305.4654 | 303.5597 | 250.7033 | 161.8003 |

*SSE, Symptomatic Skeletal Event; AIC,* *Akaike information criterion.*

**Time to First Symptomatic Skeletal Event Fit**

As for the time to first symptomatic skeletal event (SSE) Kaplan-Meier curves of BSC arm, the visual fits of the four mentioned-above parametric survival distributions was illustrated in **Figure S1B**, and the AIC statistics were detailed in **Table S1**. Although the visual fits showed that these four distributions provided similar fits for published SSE data, exponential and log-normal distributions produced higher extended tails, which implied an overestimation of SSE in the long-term. Meanwhile, based on the AIC statistics, the log-normal distribution and Log-logistic distribution provided the similar lowest AIC values. Therefore, we selected the log-logistic survival distribution for the SSE data of BSC arm in the current economic evaluation.

**Progression-free Survival Fit**

As for the progression-free survival (PFS) Kaplan-Meier curves of first-line crizotinib, the visual fits of the four mentioned-above parametric survival distributions was illustrated in **Figure S1C**, and the AIC statistics were detailed in **Table S1**. The visual fits indicated that these four distributions produced similar fits for published PFS data. We chose the log-logistic survival distribution for the PFS data of BSC arm because of its lowest AIC value.


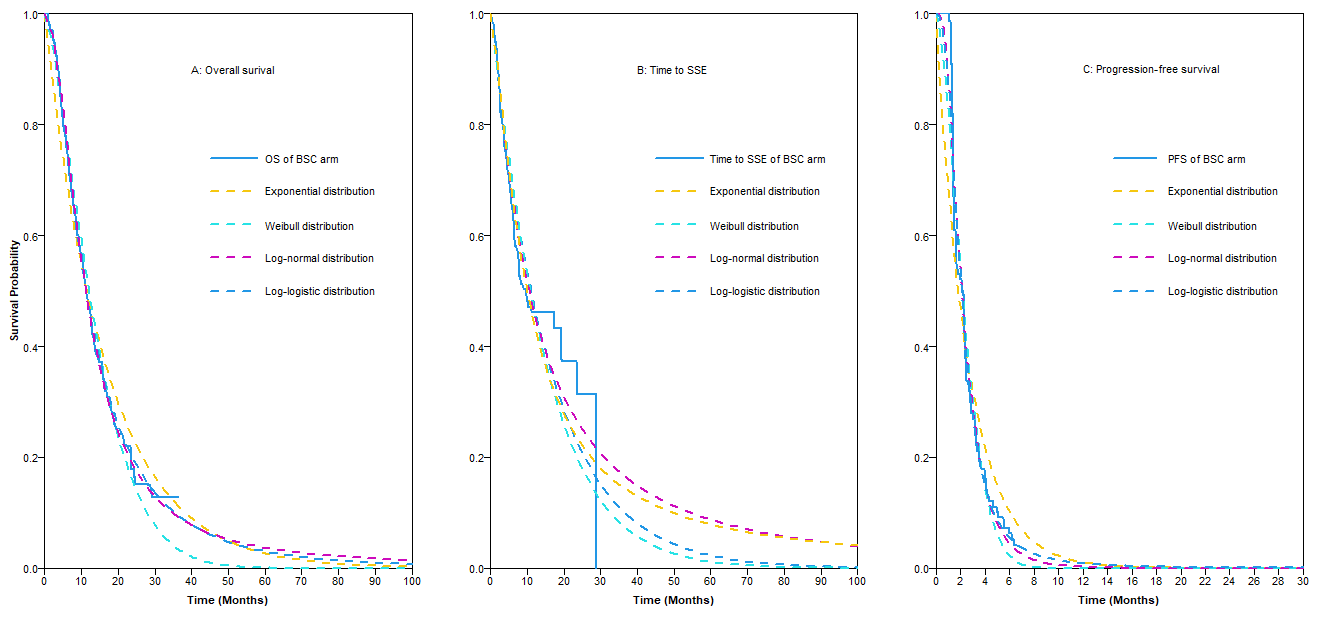


**Figure S1: Parametric survival distributions fitting for Survival probability data of BSC arm**

**Supplementary Appendix 2:** Frequency of health services

**Table S2 Frequency of health services**

| **Health service** | **Frequency** |
| --- | --- |
| Physician examination | Once every 4 weeks |
| Complete blood count | Once every 4 weeks |
| Prostatic specific Antigen testing | Once every 4 weeks |
| Testosterone levels test | Once every 4 weeks |
| Imaging CT scan (abdominal) | Once every 8 weeks in the first 24 weeks, and then once every 12 weeks thereafter |
| Bone scan | Once every 12 weeks |
